# Homeless Shelter Characteristics and Prevalence of SARS-CoV-2

**DOI:** 10.5811/westjem.2020.11.50337

**Published:** 2021-03-02

**Authors:** Massimo Ralli, Andrea Arcangeli, Aldo Morrone, Lucia Ercoli

**Affiliations:** * Sapienza University of Rome, Department of Sense Organs, Rome, Italy; † Eleemosynaria Apostolica, Primary Care Services, Vatican City State; ‡ Directorate of Health and Hygiene, Vatican City State; § Fondazione Policlinico Universitario A. Gemelli IRCCS, Department of Anesthesiology, Intensive Care and Emergency Medicine, Rome, Italy; ¶ Scientific Director, San Gallicano Dermatological Institute, IRCCS, Rome, Italy; || Tor Vergata University of Rome, Department of Biomedicine and Prevention, Rome, Italy

To the Editor:

We read with interest the article by Rebecca Karb et al^1^ titled “Homeless shelter characteristics and prevalence of SARS-CoV-2,” published in the *Western Journal of Emergency Medicine*. We appreciated the authors focusing on people experiencing homelessness, a population that has been particularly impacted by the recent coronavirus disease 19 (COVID-19) pandemic and that is more at risk of contracting COVID-19 for specific environmental and individual characteristics.^2^

In this article, the authors compared the characteristics of five different homeless shelters in Rhode Island, USA, and evaluated the prevalence of severe acute respiratory syndrome coronavirus-2 (SARS-CoV-2) infection among their residents (n = 299) using reverse transcription polymerase chain reaction (RT-PCR) nasopharyngeal swabbing. The overall prevalence across all shelters was 11.7%; however, a large difference was found between shelters, as 3/5 had no cases while two had 21.6% and 35.3% of positive cases, respectively. The authors concluded that shelters with more transient residents, higher occupation rate, admission of new residents, and absence of daily education had a higher prevalence of SARS-CoV-2 infection.^1^ In addition, the authors highlighted the importance of the population density of the neighborhood; in fact, shelters in more densely populated areas had a higher prevalence of COVID-19.^1^

Our group performed an active surveillance over a period of six months (April-September 2020) in a cohort of nearly 200 homeless persons living in shelters in the downtown area of Rome, Italy, through the medical facilities of the Eleemosynaria Apostolica, Holy See; they included the Madre di Misericordia Primary Care Center, an advanced mobile medical unit and an ambulance. In these persons, hosted in homeless shelters managed by the Eleemosynaria Apostolica, prevention strategies were adopted including the use of face masks and hygienizing gels by residents and staff, adequate social distancing, daily symptom screening and temperature checks, routine SARS-CoV-2 testing, and constant education on prevention measures to avoid contagion. Furthermore, all new admissions were tested with RT-PCR and antigen nasopharyngeal swab, rapid serology test, and quantitative antibody evaluation on whole blood before entering the shelter. The prevalence of SARS-CoV-2 infection in our cohort was approximately 2%; this rate is similar to that reported in other studies that investigated SARS-CoV-2 among homeless people living in congregate settings where similar prevention measures were implemented. Rogers et al^3^ reported an overall prevalence of 2% of 1434 persons in 5/14 homeless shelters in King County, Washington; Yoon et al^4^ found a prevalence of 2.1% of 1684 residents in 24 shelters in Atlanta, Georgia; and Bodkin et al^5^ reported a prevalence of 1% of 104 homeless persons in a shelter in Hamilton, ON, Canada.

The prevalence found in our cohort and in other studies strongly suggests that, as stated by Karb et al,^1^ symptom screening and temperature monitoring are insufficient means to reduce virus transmission in homeless shelters and emphasizes the importance of daily symptom and temperature checks, adequate physical distancing, use of individual protections, accurate testing of new residents, and daily education to methods and best practices to prevent infection spread. Furthermore, the use of frequent testing among residents and staff is also important, as infection from asymptomatic cases, not identifiable through daily symptom checks, is the predominant mode of SARS-CoV-2 spread in congregate living settings.^6^

In our opinion, these factors which are commonly adopted in shelters with a low SARS-CoV-2 prevalence, can widely contribute to maintain infection control among residents and staff and avoid outbreaks,^7^ such as the ones reported by Karb et al. and in a study by Imbert et al,^8^ where 67% of residents and 17% of staff tested positive for SARS-CoV-2 in a San Francisco, California, shelter. In this case, the prevention strategies of the shelter relied exclusively on symptomatic cases, person-based contact tracing, and symptom screening that was demonstrated as insufficient to prevent the outbreak.

It is, therefore, of utmost importance to emphasize the role of these prevention and control measures to prevent outbreaks in homeless shelters which are more vulnerable to virus transmission for their intrinsic characteristics, as well as in other group residential settings such as recovery houses, nursing homes, and other congregate living facilities hosting vulnerable populations.^9^ At the same time, shelters play a central role in assistance to homeless persons, and even temporary closures, as often reported during the COVID-19 pandemic,^10^ may have severe effects on public health management if alternative residential solutions are not promptly available.
